# Lifetime co-occurring psychiatric disorders in newly diagnosed adults with attention deficit hyperactivity disorder (ADHD) or/and autism spectrum disorder (ASD)

**DOI:** 10.1186/s12888-020-02828-1

**Published:** 2020-08-26

**Authors:** Artemios Pehlivanidis, Katerina Papanikolaou, Vasilios Mantas, Eva Kalantzi, Kalliopi Korobili, Lida-Alkisti Xenaki, Georgia Vassiliou, Charalambos Papageorgiou

**Affiliations:** 1 1st Department of Psychiatry, National and Kapodistrian University of Athens, Medical School, “Eginition” Hospital, 72-74 Vas. Sofias Ave, 11528 Athens, Greece; 2 Department of Child Psychiatry, National and Kapodistrian University of Athens, Medical School, “Agia Sophia” Children’s Hospital, 11527 Athens, Greece

**Keywords:** Attention deficit hyperactivity disorder, Autism Spectrum disorder, Co-occurrence, Lifetime psychiatric disorders, Adults

## Abstract

**Background:**

Co-occurring psychiatric disorders in adults with Attention Deficit Hyperactivity Disorder (ADHD) and/or Autism Spectrum Disorder (ASD) contribute to the burden of the healthcare and possibly to the delay of diagnosis. Aim of the study was to clinically assess the prevalence and compare lifetime co-occurring psychopathology in a sample of newly diagnosed ADHD and/or ASD adults and discuss the diagnostic challenges they pose.

**Methods:**

The lifetime prevalence rates of ten of the most frequently co-occurring psychiatric diagnoses was registered in 336 adults of normal intelligence who underwent a thorough clinical evaluation for the diagnosis of ADHD and/or ASD for the first time in their lives. Four study groups were formed: the ADHD (*n* = 151), the ASD (*n* = 58), the ADHD+ASD (*n* = 28) and the nonADHD/nonASD (NN) (*n* = 88) group.

**Results:**

At least one co-occurring psychopathology was found in 72.8% of the ADHD group, in 50% of the ASD group, in 72.4% of the ADHD+ASD group and in 76.1% of the NN group (*p* = 0.004). In all groups the most frequent psychiatric disorder was depressive disorder. The only significant difference regarding the patterns of psychiatric co-occurrence between the ADHD and the nonADHD groups (ASD and NN groups) was found for SUD (*p* = 0.001). Also, the proportion of subjects with Bipolar Disorder was significantly greater in the NN group as compared to those with ASD (*p* = 0.025).

**Conclusions:**

Our results support the high prevalence of co-occurring psychiatric disorders in adults with ADHD and/or ASD with the ASD group presenting the lowest rate. The most marked difference between the ADHD and the nonADHD groups was found for SUD. Moreover, our findings highlight the need for a thorough clinical assessment of all referred patients both in the presence and absence of ADHD and/or ASD.

## Background

Attention deficit hyperactivity disorder (ADHD) and Autism Spectrum Disorder (ASD) are both neurodevelopmental disorders with a typical onset in childhood and symptoms that usually remain persistent over time. The growing awareness of ADHD and ASD in adulthood, the increasing number of adults being referred for or requesting an ADHD or ASD diagnosis for the first time in their lives and the DSM-5 changes in diagnostic approach for ASD/ADHD co-occurrence have increased the demand for diagnostic services able to provide evidence-based assessments for this age group [1, 2]. The high frequency of co-occurring psychiatric conditions may lead to diagnostic overshadowing and contributes to the burden of the healthcare [3, 4].

The prevalence of ADHD in adults is in the range of 2–5% [5, 6]. Studies of ADHD in adult life show a striking degree of co-existing psychiatric disorders. In a cross-national epidemiologic study of ADHD from 20 countries one ADHD psychiatric comorbidity was found in 23% of cases, two in 14% of cases and three in 14% of cases. Rates were particularly high for any mood disorder (22%), any anxiety disorder (34%), substance use disorders (11%) and any behavioral disorder (15%) [5]. In a population based study of 40,000 adults, ADHD patients had a 4–9 times higher prevalence of anxiety, depression, bipolar and personality disorders, schizophrenia and substance use disorder (SUD) [7]. The strongest predictors of poor quality of life in adults newly diagnosed with ADHD was found to be the co-occurring anxiety and depression [8]. Conversely, prevalence among psychiatric patients rates of co-occurring ADHD range from 10 to 20% [9–15].

Traditionally autism has been regarded as a severe form of neurodevelopmental disorder which in many cases goes along with abnormal language, learning difficulties and low IQ. Current prevalence of ASD is estimated to be between 1 and 2% in developed countries, with recent increases primarily among those without comorbid intellectual disability [3, 16] whereas a prevalence of 1.1% with no variation across different age bands is reported in adults [17]. The high-functioning variants of the disease may run unrecognized until late in adult life [18–20] and are associated with a very high prevalence of co-occurring classical psychiatric disorders [21, 22]. Between 73 and 81% of adults with ASD meet criteria for at least one co-occurring psychiatric disorder [23–27]. Most research on adult ASD outcomes indicate depressive disorders, anxiety disorders, obsessive compulsive disorder, ADHD and personality disorders as the most common co-occurring psychiatric disorders. Multiple diagnoses are also common [28, 29]. Estimates in clinical sample-based studies are higher than population/registry based studies [22, 28, 29].

The previous diagnostic exclusionary criteria that prohibited a dual diagnosis ADHD+ASD have been amended in DSM-5 [30] leading to a growing literature on this topic. Although there has been a considerable amount of research focusing on the co-occurrence between ADHD and ASD in childhood [31–33], research data on the co-occurrence of ADHD and ASD in adulthood are still scarce. It has been considered that ADHD prevalence in ASD decreases with age and preliminary data suggest that the association between ASD and ADHD traits may be somewhat lower in adult age than in childhood/adolescence [34]. Current or past diagnosis of ADHD in ASD adult patients has been reported to be from 9.7 to 43% with most reports being within the range of 37–43% [19, 24, 26, 35–37] while 10% of a sample of patients diagnosed with ADHD in adulthood had a current or past diagnosis of ASD [38]. Epidemiological and clinical data on the psychiatric comorbidity of this particular group in adulthood are very limited. In a 20-year register study of 241,878 adults treated in a psychiatric clinic in Sweden, concomitant ADHD and ASD were seldom diagnosed. Twelve (86%) out of the 14 patients with a dual diagnosis had other diagnoses as well [39]. To our knowledge, there are only two large population-based studies [40, 41] comparing patterns of co-occurring lifetime psychiatric disorders between adults with an ASD, ADHD or ADHD+ASD diagnosis. One of them included both adults and youngsters that were too young to be diagnosed with adult-onset psychiatric disorders [40]. The other population study [41] using data from Norwegian registries assessed the prevalence of psychiatric disorders in adults with ADHD, ASD and both diagnoses and compared them to the remaining population. Adults with ADHD+ASD had severe additional psychiatric morbidity relative to adults with either ADHD or ASD alone. The prevalence rates differed significantly between adults with ASD and ADHD for all psychiatric disorders studied. The relative prevalence increase of substance use disorder was three times larger in ADHD than in ASD while the opposite was true for schizophrenia.

The above presented epidemiological and clinical research data show increased rates of co-occurring psychiatric disorders in ADHD and/or ASD patients. Of particular interest is the group of adult patients who are overlooked until adulthood. Despite the increasing awareness on the clinical presentation of the disorder, many people with ADHD are still underdiagnosed, misdiagnosed and undertreated which is partly explained by the high rates of comorbidity [2, 5, 12, 39, 42, 43]. Also, 7 to 16% of adult patients in psychiatric clinics or hospitals are finally diagnosed with ASD for the first time in their life [44, 45]. In addition, over half of undiagnosed adults with high functioning ASD initially visit general practitioners [46]. Adult patients who miss the diagnosis are more likely to be high functioning, able to camouflage their difficulties, and with prominent symptoms of psychiatric comorbidities that are thought to explain all their disturbances [39]. Being able to distinguish the core symptoms of ADHD and ASD in previously undiagnosed adults from symptoms of a co-occurrent psychiatric disorder demands not only a thorough understanding of the nature of the ADHD and ASD but also being aware of the possible patterns of co-occurring disorders.

Interview based clinical studies aiming to compare patterns of a wide range of lifetime psychiatric diagnoses in adults referred for a possible Attention Deficit Hyperactivity Disorder (ADHD) or/and Autism Spectrum Disorder diagnosis are lacking. Aim of the present study is to clinically assess the prevalence and compare lifetime co-occurring psychiatric diagnoses among adults of normal intelligence who seek to receive for the first time in their life an ADHD, ASD or ADHD+ASD diagnosis and discuss the diagnostic issues they pose .

## Methods

### Study design and participants

The study took place at the Adult Neurodevelopmental Outpatient clinic of the 1st Department of Psychiatry of the National and Kapodistrian University of Athens, Greece. It has been running since 2004 for the assessment of adults with ADHD and/or ASD and is accepting both self-referrals and referrals from other health services. Self-referrals were subjects willing to receive an ADHD and/or ASD diagnosis and were often directed to our department through patient’s associations. A waiting list based on the time of referral is formed. The assessment procedure is built on a standard diagnostic routine and is carried out by a multi-disciplinary team. During a three-year period (January 2015 – December 2017) we selected all referred adults of normal intelligence with fluent phrase speech who were assessed through the standard diagnostic routine for the first time in their life for a possible ADHD and/or ASD diagnosis. Exclusion criteria were a previous ADHD and/or ASD diagnosis, the presence of acute psychopathology requiring urgent psychiatric treatment, a current substance abuse disorder, IQ < 70 according to WAIS and a known genetic cause.

The sample consisted of 326 participants (217 men and 109 women) with mean age 31.7 years (SD = 10.4). Participants formed four groups: the ADHD group (*n* = 151, 44.8%), the ASD group *n* = 58, 17.2%), the ADHD+ASD group (*n* = 29, 8.6%) and the no neurodevelopmental disorder (NN) group consisting of the referred subjects that did not meet the diagnostic criteria for an ADHD and/or ASD diagnosis (*n* = 88, 26.1%). Table 1 shows age and sex of the four sample groups. Subjects in the NN group were significantly older as compared to the other groups and had also a greater proportion of women.

**Table 1:** Sample characteristics by study group

	ADHD or ASD Diagnosis				p
	ADHD *N =* 151	ASD *N =* 58	ADHD+ASD *N =* 29	NN *N =* 88	
Age (years), mean (SD)	31.0(10.0)	28.7(9.2)	28.8(10)	34.5(11.2)	< 0.001^a^
Sex, N (%)					
Men	106(70.2)	47(81)	19(65.5)	45(51.1)	0.001^b^
Women	45(29.8)	11(19)	10(34.5)	43 (48.9)	

^a^ANOVA; ^b^chi-square test

NN: No Neurodevelopmental Disorder

### Assessment procedure

The multi-disciplinary team that carries out all assessments in our clinic consists of psychiatrists who have extended experience in the diagnosis and treatment of Neurodevelopmental Disorders in adults trained in ADOS [47, 48], ADI-R [48, 49] and DIVA [50], clinical psychologists, a speech and language therapist, an occupational therapist and a social worker [51].

All patients and relatives have to complete an extended questionnaire that comprises demographic, educational, occupational and clinical data as well as a battery of screening instruments for ADHD and ASD, namely the Autism Quotient, the Empathy Quotient, the Barkley Adult ADHD Rating Scale-IV (BAARS-IV) [52–54]. The completed questionnaire is sent via e-mail to our clinic.

Responses to the questionnaire are then discussed with the patient and a family member when possible. Patients undergo a thorough at least two-hour psychiatric examination exploring current psychopathology and the life time presence of psychiatric disorders with the aid of the semi-structured interview Mini International Psychiatric Interview (MINI) [55, 56]. According to MINI interview, we assess the lifetime presence of the following 10 disorders: Depressive Disorder (DD), Bipolar Disorder (BD), Panic Disorder (PD), Social Phobia (SP), Obsessive Compulsive Disorder (OCD), Generalized Anxiety Disorder (GAD), Psychotic Disorder (PD), Alcohol Dependence (AD), Substance Use Disorder (SUD), and Antisocial Personality Disorder (APD). MINI is reported to have good inter-rater and test-retest reliability [55, 56]. Patients requiring urgent psychiatric treatment or present symptoms of current substance abuse are referred to special units and asked to be seen after receiving appropriate treatment.

Subsequently, during a session lasting up to 3 hours a psychiatrist assesses the existence and pervasiveness of the suspected neurodevelopmental disorders and the impairment caused by them. The DIVA is administered to all patients while the ADOS is administered to selected cases considered to be more complicated.

The final diagnosis regarding the presence of ADHD and/or ASD and co-occurring psychiatric disorders is given during a consensus meeting of the multidisciplinary team and is based on DSM-5 criteria while taking into consideration all available information. Finally, the diagnosis and possible treatment options are discussed with the patient and relatives.

The study was approved by the scientific and ethics committee of our University Department. All subjects gave written informed consent.

### Statistical analysis

Continuous variables are presented with mean and standard deviation (SD). Quantitative variables are presented with absolute and relative frequencies. For the comparison of proportions chi-square and Fisher’s exact tests were used. Analysis of variance (ANOVA) was used to compare mean values between the four groups. Bonferroni correction was used in case of multiple testing in order to control for type I error. All *p* values reported are two-tailed. Statistical significance was set at 0.05 and analyses were conducted using SPSS statistical software (version 22.0).

## Results

The number of co-occurring lifetime psychiatric diagnoses of subjects in each study group is showed in Table 2. At least one co-occurring psychopathology was found in 72.8% of the ADHD group, in 50% of the ASD group, in 72.4% of the ADHD+ASD group and in 76.1% of the NN group (*p* = .004).

**Table 2:** Number of co-occurring psychiatric diagnoses for all study groups

	ADHD	ASD	ADHD+ASD	NN	p
	N (%)	N (%)	N (%)	N (%)	
Psychopathology					
No	41(27.2)	29(50)	8(27.6)	21(23.9)	0.004
Yes	110(72.8)	29(50)	21(72.4)	67(76.1)	
Psychopathology					
No	41(27.2)	29(50)	8(27.6)	21(23.9)	0.022
1–2 Diagnoses	55(36.4)	14(24.1)	10(34.5)	39(44.3)	
> 2 Diagnoses	55(36.4)	15(25.9)	11(37.9)	28(31.8)	

Psychopathology: any co-occurring psychiatric diagnosis

**Fig. 1:**
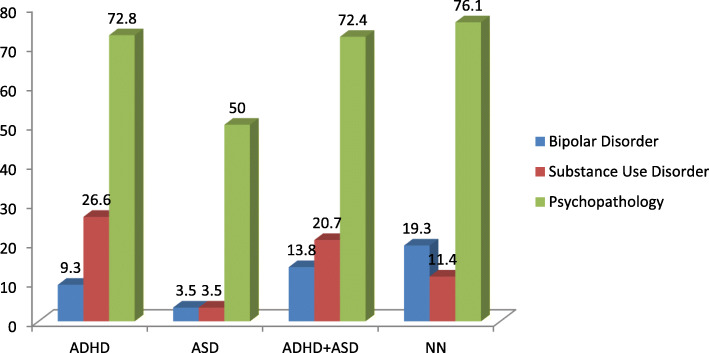
Proportion of BD, SUD diagnosis and at least one co-occurring lifetime psychiatric diagnosis

Table 3 shows co-occurring psychiatric diagnoses by study group. Significant differences were found concerning the rates of Bipolar Disorder and Substance Use Disorder. Specifically, after Bonferroni correction for multiple comparisons it was found that the proportion of subjects with Bipolar Disorder was significantly greater in NN group as compared to those with ASD. Also, the proportion of subjects with Substance Use Disorder was greater in ADHD group as compared to ASD and NN group (Fig. 1).

**Table 3:** Kind of co-occurring lifetime psychiatric diagnoses by study group

	ADHD *N =* 151 N (%)	ASD *N =* 58 N (%)	ADHD/ASD *N =* 29 N (%)	NN *N =* 88 N (%)	p
Depressive Disorder (DD)	56 (37.3)	17 (29.3)	7 (24.1)	28 (31.3)	0.462^a^
Bipolar Disorder (BD)	14 (9.3)	2 (3.5)	4 (13.8)	17 (19.3)	0.025^a^
Panic Disorder (PD)	7 (4.6)	4 (6.9)	1 (3.5)	9 (10.2)	0.374^b^
Social Phobia (SP)	5 (3.3)	1 (1.7)	3 (10.3)	2 (12.3)	0.195^b^
Generalized Anxiety Disorder (GAD)	25 (16.7)	8 (13.8)	3 (10.3)	11 (12.5)	0.746^a^
Obsessive Compulsive Disorder (OCD)	15 (10)	5 (8.6)	7 (24.1)	9 (10.2)	0.130^a^
Psychotic Disorder (PsD)	7 (4.7)	4 (6.9)	3 (10.3)	5 (5.7)	0.580^b^
Alcohol Dependence (AD)	10 (6.7)	0 (0.0)	1 (3.5)	2 (2.3)	0.121^b^
Substance Use Disorder (SUD)	40 (26.6)	2 (3.5)	6 (20.7)	10 (11.4)	< 0.001^a^
Antisocial Personality Disorder (ASPD)	11 (7.3)	2 (2.5)	4 (13.8)	7 (7.9)	0.364^b^

^a^chi-square test; ^b^Fisher’s exact test

## Discussion

Aim of the present clinical study was to examine the lifetime prevalence of co-occurrent psychiatric disorders in 326 adults of normal intelligence referred for an ADHD or ASD diagnosis for the first time in their lives. In the four study groups formed (ADHD, ASD, ADHD+ASD, NN) lifetime prevalence rates of ten of the most frequently co-occurring psychiatric diagnoses were registered.

Among the probands that received an ADHD and/or ASD diagnosis the ADHD only and the ADHD+ASD groups presented the highest rates of co-occurring disorders. In both groups approximately three quarter of the patients had at least one co-occurring psychiatric disorder and of those almost half received three or more diagnoses. Our findings are in line with the majority of data available in the literature for newly diagnosed [57] or previously diagnosed ADHD adult patients [7, 14, 58] and support the additive effect of ADHD on psychiatric comorbidities [40, 41]. Half of the ASD only patients had a co-occurring psychiatric diagnosis. This percentage is lower to the rates 73–81% reported in previous clinical studies [23, 24, 26, 27] or the pooled prevalence of 60.5% derived from a meta-analysis when including only studies of adults with ASD based on clinical interviews [29]. This is likely explained by the fact that in previous studies ASD samples included ADHD+ASD patients as well.

Interestingly, subjects in the NN group had equally high rates of psychiatric disorders. Although some differences in demographic parameters were found in this group, most importantly it is their psychopathological characteristics that pose diagnostic challenges. It might be that with the increased awareness of adult ADHD and ASD, some patients with resistant to treatment psychopathological conditions attribute their symptoms to a neurodevelopmental disorder.

In the ADHD group the highest rates of co-occurring disorders were the ones related to Depressive Disorder (DD) and Substance Use Disorder SUD), in the ASD group to Depressive Disorder (DD) and Generalized Anxiety Disorder (GAD), in the ADHA+ASD group to Depressive Disorder (DD) and Obsessive Compulsive Disorder (OCD) and in the NN group to Depressive Disorder (DD) and Bipolar Disorder (BD). The only significant difference regarding the patterns of psychiatric co-occurrence between the ADHD and the nonADHD groups (ASD and NN groups) was found for SUD. The low prevalence rate of SUD in adults with ASD and the marked difference between ADHD and ASD are consistent with previous studies in adults [29, 41]. A significant difference was also noted in BD which was more common in the NN group compared to the ASD group. Since it was a clinical study the limited number of patients did not allow us to find other distinct patterns of co-occurring psychiatric disorders in the ADHD, ASD and ADHD+ASD groups, as it was found in a previous population study [41]. Also, since there are no clinical studies comparing patterns of co-occurring lifetime psychiatric disorders, comparison of patterns can be made only with population studies [40, 41). It should be noted though, that comparison groups in these studies consisted of general population subjects and differ to our NN group which includes clinically referred patients that did not meet the criteria for an ADHD and/or ASD diagnosis.

Below, we discuss our findings regarding the lifetime prevalence of each co-occurring psychiatric diagnosis in all groups in relation to data from clinical and epidemiological studies and we highlight some of the diagnostic challenges clinicians have to face with adults referred for a possible ADHD or ASD diagnosis.

### Depressive disorder (DD)

The most commonly reported psychiatric disorder in all groups was depressive disorder (37.3% in ADHD, 29.3% in ASD, 24.1% in ADHD+ASD and 31.3% in the NN group). Although being close to the lower rate, our findings on patients with ADHD are consistent with the 32–60% lifetime prevalence of co-occurring depression reported in previous studies [11, 59]. Also, the rate of depression in the ASD group lies in between the pooled estimates of 12% [22] and 37% [21] found in meta-analyses of previous studies including ASD population and is lower to the rate 53% of Mood Disorder (including BD) in de novo diagnosed adults with ASD, with Asperger Syndrome and PDD NOS [24]. In the population study using data from Norwegian registries the prevalence of Major Depressive Disorder (MDD) was 24.2% for adults with ADHD, 14.3% for adults with ASD and 20% for ADHD+ASD [41] somewhat lower to the rates found in our sample. This was to be expected since in population studies, milder cases might have not be registered especially in people with ASD who are less likely to seek help for a mood disorder. Although the combination of both diagnoses would possibly imply greater difficulties, the co-occurrence of ASD and ADHD did not increase the rate of DD in our sample. It might be that the presence of ASD in subjects with ADHD makes more difficult the expression of depressive feelings.

It is of particular note that the NN group had a high ratio of DD. Overlapping symptoms such as decreased attention, memory and concentration have to be taken in consideration in the differential diagnosis among DD and ADHD. Also, diagnostic overshadowing together with difficulties in describing low mood are difficulties that a clinician has to overcome when conducting diagnostic interviews for co-occurring DD in ASD patients.

### Bipolar disorder (BD)

The rate of lifetime co-occurring BD in the ADHD group was 9.3%., in the ASD group 3.5%, in the ADHD+ASD group 13.8% while almost one fifth of the NN group (19.3%) received a BD diagnosis.

High rates of lifetime prevalence of co-occurring ADHD among BD have been consistently reported in adults ranging from 9.5 to 25% [11, 59–62]. Conversely, studies on the prevalence of BD in adults with ADHD are scarce and controversial showing rates from 5.1 to 47% [63–65].

The diagnosis of ASD is also associated with an increased risk of BD [66]. Systematic reviews and meta-analyses show inconsistent results, namely an overall pooled estimate of BD in ASD patients of 5% [22] or 21.2% [29] and a BD prevalence of 6–21.4% in a sample of adults with Asperger syndrome [67]. The nationwide, population based study in Norway reported a prevalence rate of 7.8% for adults with ADHD, 5.3% for the ASD group and 9.4% for the ASD + ADHD group [41].

Our results are close to the lower rates of BD co-occurrence found in previous studies of ADHD patients and even lower compared to previous ASD adult samples while the ADHD+ASD group although more impaired still shows a lower to the Norwegian population study prevalence. Discrepancies might be attributed to the different study design and the fact that our sample consists of newly diagnosed adult patients, often self-referred, who are expected to be less impaired compared to those diagnosed earlier in life.

The finding that the rate of BD in the NN group was so high is very interesting, supporting diagnostic overshadowing. Symptoms of a maniac or hypomaniac episode and emotional lability can be easily attributed by patients with BD or clinicians to ADHD symptomatology, leading them to ask for an ADHD assessment. Several of the diagnostic criteria for ADHD and bipolar disorder, such as increased activity, distractibility, irritable mood, talkativeness, are similar. A thorough clinical evaluation including past psychiatric and developmental history that would clarify features such as age of onset, persistent or episodic display of symptoms is necessary when examining adults referred for a neurodevelopmental disorder diagnosis [9].

### Panic disorder (PD), social phobia (SP), generalized anxiety disorder (GAD)

Our findings on the co-occurrence of PD (4.6%) and GAD (16.7%) in ADHD patients were consistent with those presented in previous studies [68–70]. On the contrary, the lifetime prevalence of social phobia (3.3%) in our ADHD sample was lower to the 11–38% rate reported in most studies [68–71].

In the ASD group the prevalence of PD (6.9%) and GAD (13.8%) was in-between the rates reported in other studies ranging from 1 to 18% for PD and 11.8–26% for GAD [21, 36, 72]. Surprisingly low was the prevalence of SP (1.7%) compared to rates in previous clinical reports ranging from 12.4 to 56% [21, 26, 36, 72, 73] and 3.95% in a recent population study [74].There is a considerable overlap between the contact and communication problems of ASD and SP subjects including characteristics such as social withdrawal and being quiet in social situations [75, 76].

There are some possible reasons explaining at least to some extend the low rate of SP in both our ASD and ADHD samples. First, it might be that either we were trapped in the diagnostic overshadowing between ASD and SP or that other researchers considered core symptoms of ASD as social anxiety. The lack of standardized tools measuring anxiety in ASD individuals makes more difficult for clinicians to disentangle symptoms of social phobia from ASD symptoms. Second, our ASD sample consisted of high functioning individuals, newly diagnosed and possibly less socially impaired. Finally, after the release of DSM-5, clinicians are allowed to give a dual diagnosis of ASD and ADHD and some patients with both ADHD and ASD symptoms who in the past would receive an ADHD+social phobia diagnosis can know receive an ADHD+ASD diagnosis. We suggest therefore that in previous reports there might be an overdiagnosis of SP in ADHD patients.

In the ADHD+ASD group all three anxiety disorders presented a prevalence of approximately 10%. The increased rate of SP in our dual diagnosis group suggests that even if some formerly ADHD+SP cases are now classified as ADHD+ASD cases SP remains a true co-occurrent disorder in probands with ADHD+ASD. Regardless, the nature of social anxiety in ASD and/or ADHD is an issue that has to be further investigated [77].

The NN group showed elevated rates of all three anxiety disorders. Restlessness-psychomotor agitation, concentration difficulties, decreased attention, increased distractibility, mood swings, and anger outbursts are symptoms that overlap between ADHD and anxiety disorders [78, 79]. Differentiation is even more difficult with GAD which has a much more chronic course and early onset age and more overlapping characteristics with ADHD. Also, the similar to ASD surface-level presentation of social avoidance of people with SP can lead them to seek a possible ASD diagnosis.

### Obsessive compulsive disorder (OCD)

The rate of lifetime co-occurring OCD was similar in the ADHD and the NN groups (approximately 10%), somewhat lower in the ASD group (8.6%), with a 2.5-fold increase in the ADHD+ASD group (24.1%).

Our results on the ADHD group are in accordance with previous findings showing a 7–13% prevalence of OCD in adults with ADHD [80]. In adults with ASD the OCD prevalence reported by previous researchers is variable with pooled prevalence rates from 9% [22] to 22% [21]. In newly diagnosed ASD adult patients current OCD was the single condition identified significantly more frequently compared to non ASD patients [36]. In a recent population study the OCD prevalence in ASD patients was 3.85% [74] which might be an underestimation since it derives from a population study where overshadowing effects between ASD and OCD are very probable.

In our study the highest rate was noted in the dual diagnosis group. Although the small sample did not allow a statistically significant difference among groups, a possible additive effect of the presence of both ADHD and ASD on the occurrence of OCD cannot be ruled out. To our knowledge there are no previous studies exploring the co-occurrence of OCD in ADHD+ASD adults.

The elevated prevalence of OCD in the NN group underlines the need for a thorough clinical assessment of the referred individuals. The Executive Overload model [81] suggests that deficits in attention and executive function in OCD patients may result in a phenotypical expression that may resemble ADHD symptoms. Also, because of the diagnostic similarities between ritualistic and repetitive behaviours in ASD and obsessions and compulsions in OCD, differential diagnosis might become a challenge. In his seminal work, Kanner in 1943 already suggested that the core symptoms of autism are anxiety-driven, assuming that the autistic child’s behavior is governed by an anxious and obsessive desire for maintenance of sameness.

### Psychotic disorder (PsD)

The rate of lifetime co-occurring PsD in the ADHD group was 4.7% which is comparable to the previously reported co-occurrence of PsD and ADHD which can be up to 11%, especially when ADHD is of inattentive type [82] or associated with dysphoric mood [83] and persistent cannabis use and self-harm [84, 85].

The rate of 6.9% co-occurrence of PsD in our ASD group is among the lowest reported in the literature. Rates of co morbid PsD in ASD population, possibly more with atypical affective disturbances [86], present a wide range and vary from 3.6 to 60% [87]. Also, in a recent umbrella review, the prevalence of schizophrenia spectrum and other psychotic disorders reported in eight systematic reviews and meta-analyses including adults with ASD ranged from 4 to 67% [25]. The study design and the characteristics of our sample might explain the low rates found in our study.

In the dual diagnosed group we could hypothesize that the additive effect of the neurocognitive and developmental deficits of ADHD and ASD would increase the rate of a lifetime diagnosis of a psychotic disorder. The only study assessing Schizophrenia Spectrum Disorder (SSD) in adults with ADHD+ASD, is the Norwegian population study [41] which confirmed the increased rates of SSD in this group compared to the ADHD group. In our study, although the rate in the ADHD+ASD group was twice the rate of the ADHD group and presented a 50% increase compared to the ASD group, the sample was not large enough to point out any statistically significant differences between groups.

The rate of 5.7% in the NN group highlights the multiple phenotypic commonalities between ADHD, ASD and PsD and the risk for a misdiagnosis, especially in unidentified ASD and ADHD adults. The attentional deficits, hyperactivity features, emotional dysregulation, and disorganized behavior that might accompany psychosis may not be “true” ADHD. Also, impairments in social interaction and communication, lack of emotional response, poverty of speech, withdrawal from reality accompanied by extended fantasy and catatonic features are common features of ASD and PsD that can complicate the diagnosis of adults referred for an ASD diagnosis.

### Substance use disorders (SUD) and alcohol dependence (AD)

In our study SUD was the only co-occurring psychiatric disorder whose prevalence significantly differed between ADHD, ASD and NN groups. The rate in the ADHD group was almost four times higher than in the ASD group (26.6% vs 3.5%) while in the ADHD+ASD group the rate was comparable to the rate of the ADHD group (20.7%). Rates of AD were much lower in all groups and in fact there were no reports of co-occurring ASD and AD cases. In the NN group rates of SUD were significantly lower to the ADHD group but still substantial (11.4%).

Our ADHD findings are in line with previous reports of SUD prevalence in ADHD ranging from 12 to 58% [11, 57, 63, 64, 88, 89]. On the other hand the prevalence of co-occurring AD was at the lower end of the 6–44% rates reported among ADHD patients in previous studies [11, 63, 64]. Estimates of SUD and AD in ASD individuals are contradictory. In all previous reports, rates of SUD were higher to the ones found in our study and a null rate of ASD-AD co-occurrence is not reported elsewhere. The rate of a lifetime alcohol related misuse disorder among normal intelligence patients with de novo diagnosed ASD was reported to be 12% [24] and in a sample of patients with Asperger Syndrome, SUD and alcohol misuse presented a prevalence of 7% each [90]. In a systematic review and meta-analysis of 16 studies of adults with ASD the pooled prevalence of SUD including AD was 8.3%. It has to be noted though that in all previous reports a high percentage of cases had a co-occurring ADHD diagnosis. Nevertheless, even in ASD only samples the prevalence remains high compared to our findings; namely, SUD including AD was 10.9% in adults with ASD and normal intelligence [91] while a 30% prevalence of SUD was reported in another ASD only adult population [89]. A Swedish population-based cohort study found that the ASD diagnosis doubled the risk for SUD adults of normal intelligence who do not represent a co-occurring ADHD diagnosis [92].

The low rates of AD and SUD in our ASD sample apart from the existence of a separate ADHD+ASD group can be also attributed to the exclusion of patients with a current co-occurring AD or SUD. It might also reflect cultural differences among various countries. The same reasons could explain the low rate of AD in our ADHD patients.

In our sample the co-occurrence of ADHD+ASD increased the risk for a lifetime diagnosis of SUD in comparison to solely ASD which is in accordance with the rates of AD and SUD found in ASD samples including ADHD patients. In the Norwegian population based study [41] where ADHD+ASD patients formed a separate group as in our study, the rates were lower to our study which is expected in a population study. The pattern though of the prevalence rates of SUD in all groups was in accordance to the one found in our sample, namely 7.8% for ADHD patients, 2.1% for ASD patients and 4.2% for ADHD+ASD patients.

The above support the notion that SUD and alcohol related disorders are lifetime diagnoses that have to be taken in consideration when assessing individuals referred for an ADHD and/or an ASD diagnosis in adults. The personality trait novelty-seeking is strongly related to SUDs, alcohol related disorders and ADHD personality characteristics [93]. They share impulsivity, aggression, underachievement, less schooling, poor self-esteem, social skills deficits, antisocial behaviors and depression [94]. These characteristics could explain both the difference in the prevalence of SUD between ADHD and ASD groups and the fact that a substantial rate of adults with SUD is referred for a possible ADHD diagnosis even in the absence of ADHD. Also, examining the common behavioral model of SUD/Alcohol misuse with the core symptomatology of ASD, both share the repetitive pattern and the persistence, the high levels of anxiety disorders as well as the difficulties in social interaction which might be a diagnostic challenge when assessing adults referred for an ASD diagnosis.

### Antisocial personality disorder (ASPD)

The rate of lifetime co-occurring ASPD in the ADHD group was 7.3%, in the ASD group 2.5%, in the ADHD+ASD group 13.8%, and in the NN group 11.4%.

In previous studies 12–17% of ADHD adults [63, 64, 95] received a co-occurring ASPD diagnosis. Antisocial behavior is related to hyperactivity and impulsivity [96] and ADHD alone is considered a strong predictor of ASPD [97]. As already mentioned for previous psychiatric disorders, the lower rate in our study could be related to our study design and sample characteristics.

Our ASD findings are in line with previous reports showing low rates of ASPD in ASD individuals. In de novo diagnosed adults with ASD, 3% of ASD patients received an ASPD diagnosis with 43% of ASD patients having a co-occurring ADHD [24] while in a sample of young adults with Asperger syndrome [90] none received the ASPD diagnosis.

In the Norwegian population study the prevalence rates of ASPD were higher to the rates found in our study. Although, the sample is much smaller and differences do not reach a statistical significance our findings follow the same pattern, namely the highest rate is noted in the dual diagnosis group followed by the ADHD group while the ASD group presents a low rate.

The elevated prevalence of ASPD in the NN group might reflect the confusion between ASD and ASPD that arises owing to the “lack of empathy”, although empathy profiles in the two disorders seem to be the opposite (reduced cognitive empathy in ASD vs reduced affective empathy in ASPD) [98, 99, 100].Also, the well established association of ADHD and ASPD might lead clinicians to refer individuals with ASPD for an ADHD assessment.

## Strengths and limitations of the study

Very few studies have assessed and compared co-occurring lifetime psychiatric disorders in newly diagnosed adults with ADHD, ASD and ADHD+ASD or nonADHD /ASD probands. The fact that the sample is homogeneous in many aspects namely in terms of functioning and intellectual ability, absence of a previous ADHD and ASD diagnosis and of acute psychopathology, can also be considered as a strength of the study. Moreover, the diagnosis was made in consensus by experienced psychiatrists in developmental disorders trained in structured interviews for ADHD and ASD, working in a university psychiatry clinic and supplemented with psychologists for the assessment of possible intellectual disability. On the other hand some of the strengths could also be considered as limitations of the study. We cannot generalize our findings to the entire ADHD and ASD populations. The exclusion of patients with current AD and SUD and patients requiring urgent psychiatric treatment might have biased all groups to present less co-occurring disorders and may account for some of the differences found in our study compared to others. Further, since it was a clinical study including all referred patients during a three-year period, sample sizes were disproportionate and males outnumbered females as expected according to already known clinical data [2, 16]. The generalizability of the results is also limited by the fact that our NN group consists of clinically referred subjects that did not meet the criteria for an ADHD and /or and ASD diagnosis and a general population group was not included. The use of the above group, though, offers very important information on the diagnostic issues that are posed during the assessment of ADHD and ASD in adults. It is also important to note that a standardized interview for the diagnosis of ASD was nor used in all cases. Nevertheless, we have to consider first that assessment was based on our standard clinical practice emphasizing on a thorough and multidisciplinary clinical assessment and second that research data cautiously confirm the accuracy of ADOS-2 Module 4 in adults while ADI-R might not be reliable in adults without intellectual disability [100]. Another limitation of the study is the fact that only ten psychiatric disorders are assessed when administering the MINI. For instance, Borderline Personality Disorder which shares behavioural impairments and shows a high co-occurrence with ADHD is not assessed by the MINI. On the other hand, the fact that MINI does not query for externalizing disorders, most notably ADHD, was not a problem in our study since ADHD was separately diagnosed. Finally, a larger number of patients could possibly allow the emergence of more significant differences in the patterns of co-occurring psychiatric disorders between ADHD, ASD and ADHD+ASD groups.

## Conclusions

In diagnosing and treating adults with ADHD and ASD clinicians should be able to overcome difficulties related both to the high frequency of co-occurring psychiatric conditions and to the symptom overlap between the two disorders. The co-occurrence of ADHD in ASD patients seems to significantly increase the total rates of psychopathology while the most marked difference between the ADHD and the nonADHD groups was found for SUD. Interestingly, a significant proportion of adults referred for a possible ADHD or ASD do not meet criteria for any of those two neurodevelopmental disorders and meet criteria for other mental health problems. The detection of Bipolar Disorder seems to be of particular importance since almost one fifth of the nonASD and nonADHD patients suffered from it. Therefore, in order to assess for possible ADHD and/or ASD in adults there is a need for a thorough clinical assessment by an experienced in the neurodevelopmental disorders clinical team able to provide the correct diagnosis and treatment which are essential in changing the prognosis.

## Data Availability

The datasets used and analyzed during the current study are available from the corresponding author on reasonable request.

## Abbreviations

AD: Alcohol Dependence
ADHD: Attention Deficit Hyperactivity Disorder
ADHD+ASD: Co-occurrence of ADHD and ASD
ADI: Autism Diagnostic Interview
ADOS: Autism Diagnostic Observational Schedule
ANOVA: Analysis Of Variance
ASD: Autism Spectrum Disorder
ASPD: Antisocial Personality Disorder
BD: Bipolar Disorder
DD: Depressive Disorder
DIVA: Diagnostic Interview for ADHD
GAD: Generalized Anxiety Disorder
IQ: Intelligence Quotient
MDD: Major Depressive Disorder
MINI: Mini International Psychiatric Interview
NN: No Neurodevelopmental Disorder Group
OCD: Obsessive Compulsive Disorder
PD: Panic Disorder
PsD: Psychotic Disorder
SD: Standard Deviation
SP: Social Phobia
SPSS: Statistical Package for Social Sciences
SSD: Schizophrenia Spectrum Disorders
SUD: Substance Use Disorder
WAIS: Wechsler Adult Intelligence Scale
